# Comparison of Costs of Home and Facility-based Basic Obstetric Care in Rural Bangladesh

**DOI:** 10.3329/jhpn.v28i3.5558

**Published:** 2010-06

**Authors:** J. Borghi, N. Sabina, C. Ronsmans, J. Killewo

**Affiliations:** ^1^ Health Policy Unit, London School of Hygiene & Tropical Medicine, Keppel Street, London WC1E 7HT, UK; ^2^ Public Health Sciences Division, ICDDR,B, GPO Box 128, Dhaka 1000, Bangladesh; ^3^ Infections Disease and Epidemiology Unit, London School of Hygiene & Tropical Medicine, Keppel Street, London WC1E 7HT, UK; ^4^ Muhimbili University College of Health Sciences, PO Box 65015, Dar es Salaam, Tanzania

**Keywords:** Comparative studies, Cost-benefit analysis, Healthcare costs, Maternal health, Obstetric care, Bangladesh

## Abstract

This study compared the costs of providing antenatal, delivery and postnatal care in the home and in a basic obstetric facility in rural Bangladesh. The average costs were estimated by interviewing midwives and from institutional records. The main determinants of cost in each setting were also assessed. The cost of basic obstetric care in the home and in a facility was very similar, although care in the home was cheaper. Deliveries in the home took more time but this was offset by the capital costs associated with facility-based care. As use-rates increase, deliveries in a facility will become cheaper. Antenatal and postnatal care was much cheaper to provide in the facility than in the home. Facility-based delivery care is likely to be a cheaper and more feasible method for the care provider as demand rises. In settings where skilled attendance rates are very low, home-based care will be cheaper.

## INTRODUCTION

Skilled attendance during pregnancy and childbirth has been promoted as the single most effective means of successfully reducing maternal mortality in poorer countries, with the maximum coverage being most desirable (1). Yet, in many countries, a large number of women continue to deliver in the home without the presence of an adequately-trained or equipped care provider. In addition, a few women have adequate prenatal or postnatal care. In Bangladesh, for example, only 12% of women deliver with a skilled attendant, only one in five women makes three or more antenatal visits (2), and very few have any postnatal care (3).

The Government of Bangladesh has made it a national priority to increase skilled attendance at delivery to address the high levels of maternal mortality (4). For non-complicated deliveries, care can be organized in various ways, and trained staff can attend women in the home or in a health facility. The question of where deliveries should take place is a topic of continued debate (5, 6).

In Bangladesh, efforts have been made to upgrade essential obstetric care facilities (7) and to mobilize communities to use health services, by reducing financial and distance barriers using vouchers (8). Starting in early 2003, the Government, with support of the World Health Organization and the United Nations Population Fund, has trained basic healthcare workers (female health assistants and family welfare assistants) to conduct delivery and provide postnatal and neonatal care in the home (9). A few developing countries, including Indonesia (10) and Bangladesh (11), have tried to promote deliveries in the home with trained midwives.

Little is known regarding the relative efficiency and effectiveness of providing basic obstetric care to women in the home compared to in a facility. To date, no studies investigated the relative cost of providing birth-related assistance in the home or in a health facility in developing countries. Two studies have explored the comparative cost of home versus hospital care in Western Europe for postnatal care (12) and for high-risk pregnancies (13). Numerous studies have also considered the comparative cost of home care versus hospital care for other services, such as care of the elderly (14), directly-observed therapy (DOT) for patients with tuberculosis (15), haemodialysis (16), care of serious mental illness (17), and administration of intravenous immunoglobulin (18). Only one of these studies was conducted in a developing-country setting (15). All found that home-based care was more cost-effective than facility-based care, except for one which found no difference.

The Matlab demographic surveillance site is a rural area of Bangladesh, located 60 km southeast of the capital—Dhaka—having a population of 100,000. Within this setting, over a 15-year period, two approaches to providing care during delivery were implemented sequentially: (a) attendance of births in the home by skilled care providers and (b) attendance of births in health facilities (19). This setting, therefore, provided a unique opportunity to explore the relative costs of these two methods of service provision in a developing-country setting. The aim of this study was to estimate and compare the costs of providing basic obstetric care in the home and in a low-level health facility.

## MATERIALS AND METHODS

### Study site

Since 1966, the International Centre for Diarrhoeal Disease Research, Bangladesh (ICDDR,B) has maintained registration of all births, deaths, and migrations in the Matlab area. The area comprises four subcentres (small health centres), which were upgraded to perform basic obstetric care between 1996 and 2001, and a clinic in Matlab town which can manage normal and forceps deliveries. All are funded, staffed, and maintained by ICDDR,B. Complicated cases are referred either to the government district hospital in Chandpur or to private hospitals, the number of which is growing.

### Overview of home- and facility-based approaches to basic obstetric care

During the period of home-based maternity care between 1987 and 1996, two midwives were posted in each of the four subcentres and were encouraged to attend as many deliveries in the home as possible, to organize and accompany referrals to the clinic in Matlab town, and to provide antenatal and postnatal care in the home (11). In response to a call by a family member, they visited the home of the woman by rickshaw or by country-or speedboat, depending on the season (20). Antenatal care was provided routinely during visits to the community, and postnatal care was provided within 48 hours of delivery.

The shift to facility-based care from 1996 onwards involved building and equipping a delivery and post-delivery room in each subcentre. Women travelled a maximum distance of three kilometres to the subcentre for antenatal, delivery and postnatal care. The care provided in the facilities was very similar to that provided in the home. In the event of complications, women were referred and accompanied by a midwife to the ICDDR,B clinic in Matlab town.

### Costing methods

We adopted a care provider (ICDDR,B, donor, and the Ministry of Health and Family Welfare) perspective to estimate costs. We considered the timeframe of pregnancy and the immediate postpartum for the analysis of costs and estimated the unit costs of antenatal and postnatal care provision and basic obstetric care for uncomplicated vaginal deliveries. The unit cost of referral care either at the Matlab clinic or at higher-level public or private hospitals was not included as it was assumed that the cost would be the same, irrespective of where the woman was referred from (home or subcentre). Although transport costs might differ, these were incurred by the patient and were outside the scope of the present study. Data on costs were collected in 2002 and 2003. The methods of collecting data on cost are described below.

An ingredients approach, whereby quantities of each of the inputs are first identified and then prices are attached, was employed to estimate the majority of recurrent costs (salaries, drugs and medical supplies, and fuel) (21). Capital items (equipment, building, and land) and overhead (utilities and maintenance), which are shared between different healthcare services, were allocated based on service-use. For example, if a room was used for providing antenatal services and care to children aged less than five years, the value of the room in terms of land, utilities, and maintenance would be shared between these two services and allocated to antenatal care based on the relative number of visits.

Interviews were conducted with all eight midwives and five boatmen/rickshaw-pullers to estimate the average time spent travelling to and attending women, typical drugs and medical supplies used for care in the home and in the subcentre, equipment used for deliveries in the home (for midwives), and consumption of fuel (for boatmen). The questionnaire was translated into Bangla and back-translated into English. Four female data collectors were trained in the use of the questionnaire during a one-week period. Observation of midwife practices and analyses of current medical records were not possible due to changes in the medical protocol for maternity services since 2001. An inventory of the subcentres provided information on the equipment used for deliveries in the facilities.

Data on salaries, costs of building and construction work carried out to upgrade health facilities, costs of vehicles, and prices of fuel were obtained from the financial records of ICDDR,B. The financial records of the health facility were used for estimating expenditure on utilities and facility maintenance. Market prices were used for valuing equipment and the land on which facilities were constructed. Land was donated for three subcentres and valued at the depreciation rate, or interest that would have been accumulated if the money had been invested. The prices of drugs and medical supplies were obtained from a yearly publication (22). Capital costs were annualized depending on the expected length of life of the item.

Given the difficulty of obtaining retrospective data on some variables, a number of assumptions were made to complete the gaps:

- The drugs and medical supplies used in the home were assumed to be the same as those used in the facility, with the addition of a safe delivery-kit for deliveries in the home. There was no clear indication from the midwives that there were systematic differences in drugs and supplies used.
- In terms of time, an inpatient admission was assumed equal to 59 times an outpatient visit which was based on the average time spent on deliveries compared to antenatal visits as reported by the midwives we interviewed. This factor was used for allocating the overhead costs of the health facility.
- For transport, in three of the four subcentres, midwives were assumed to use rickshaws during the dry season to attend deliveries in the home (58% of the year) and a country-boat in the wet season (42% of the year). In the fourth subcentre, a country-boat was used only for 32% of the year, with a speedboat being used for 10% of the year. This was based on an assessment made by the staff working at Matlab.
- It was assumed that visits for antenatal and postnatal care took the same amount of time. There was no clear indication from the midwives that there were systematic differences in the duration of the visits.
- It was assumed that the equipment and furniture in the delivery and postnatal rooms were only used for deliveries. Equipment and furniture in the examination room were used for all outpatient visits, including antenatal and postnatal care. This was based on a discussion with the facility in-charges.

Calculations of costs were undertaken using Microsoft Excel software. All the costs are presented in Taka as in 2001 (US$ 1=Tk 53.96). Costs are expressed as medians in the main analysis with the minimum and maximum values to provide a range of uncertainty. Costs represent the unit cost of providing the service.

A series of one-way threshold analyses were conducted to ascertain the degree of increase or reduction in key parameters required to make the difference in cost between basic obstetric care in the home and in the subcentre equal to zero (23). We also considered the effect of variations in staff salaries, time spent attending women, and the value of equipment and building. Given the potential for economies of scale for deliveries in the subcentres, we considered the effect of an increase in the number of deliveries in the subcentre on the cost of care.

## RESULTS

### Health service-use

The numbers seeking basic obstetric care in the home reached a peak of 531 (20%) in 1992, or 133 births in the home per year per pair of midwives (24). The numbers seeking care in a subcentre reached a peak of 454 (16%) in 2001 or 133 births per subcentre per year. Although there is an increasing trend over time, the trend does not increase significantly for facility-based births.

### Unit costs

The reported time spent attending women during delivery was greater in the home than in the subcentre (9 vs 4 hours) (Table 1). While the boatman or rickshaw-puller would wait for the midwife throughout the period of deliveries in the home, under the policy of deliveries in the subcentres, they served as cleaners, spending an estimated hour per delivery. For antenatal and postnatal care, the reported time spent by midwives attending women in the home was much more than that spent in the subcentre (54 vs 34 minutes).

**Table 1.** Median time spent by service provider (minimum and maximum values in parentheses)

Category of service provider	Home			Subcentre
	Travel time–return (hours)	Time spent with woman (hours)	Total hours/woman	Time spent with woman (hours)
Delivery care				
Midwife	3 (1–5)	7 (3–10)	9 (3–15)	4 (3–7)
Rickshaw-puller/boatman/cleaner	3 (1–5)	7 (3–10)	9 (3–15)	1
Antenatal/postnatal care
Midwife	3 (1–5)	0.9 (0.5–1.0)	4 (2–6)	0.56 (0.50–0.58)
Rickshaw-puller/boatman	3 (1–5)	0.9 (0.5–1.0)	4 (2–6)	0

The average costs of basic obstetric care in the home and in a subcentre were similar, although the costs of care in the home were lower (by Tk 76) (Table 2). The higher staff costs in the home were compensated by higher equipment and building costs in the subcentre. The time spent by midwives attending deliveries in the home, the number of deliveries in the subcentre, and costs of construction were the most influential parameters determining the difference in cost between the two approaches (Table 3). For example, the number of deliveries per subcentre would only need to increase from an average of 114 to 136 per year (by 22.5%) for there to no longer be any difference between the cost of basic obstetric care in the home and in a subcentre. Similarly, if midwives spent an extra 1.72 hours attending deliveries in the home than originally assumed (8.67 hours) (a 20% increase), there would no longer be a difference in cost between basic obstetric care in the home and in a subcentre.

**Table 2.** Unit costs (Tk) of delivery in the home and in the facility

Category of cost	Delivery in home (1)	Delivery in facility (2)	Difference (2-1)
	Median (min-max)		
Staff			
Midwife	246 (88–412)	105 (71–189)	-141
Rickshaw-puller/boatman/cleaner	150 (26–223)	15	-135
Total staff	396 (114–635)	120 (86–204)	-276
Drugs	70 (21–147)	70 (21–147)	0
Medical supplies	169	119	-50
Fuel	51 (0–51)	0	-51
Utilities and maintenance	0	58 (32–83)	58
Total recurrent	686 (303–1,001)	366 (257–552)	-320
Equipment	15 (12–19)	163 (80–352)	148
Vehicle	32 (0–87)	0	-32
Building and land	0	280 (216–363)	280
Total capital	47 (12–106)	443 (296–716)	396
Total	732 (315–1,107)	808 (553–1,268)	76

Max=Maximum;

Min=Minimum

**Table 3.** Threshold values for key parameters

Parameter included	Initial value	Threshold value	% change to reach threshold value
Salaries—midwife (Tk per hour)	28	37	+30
Rickshaw-puller (Tk per hour)	16	21
Time (hours) spent travelling to and attending deliveries in home	8.67	10.39	+20
Time (hours) spent attending deliveries in a facility	3.69	1.00	-73
Annual value (Tk) of equipment per facility	18,436	7,374	-47
Annual value (Tk) of construction per facility	38,593	27,787	-27
Allocation factor for inpatient versus outpatient admissions (1 inpatient=X outpatients)	X=59	X=23.5	-47
Increase in rate of facility-based delivery per year	114	136	+22.5

**Fig. 1.**
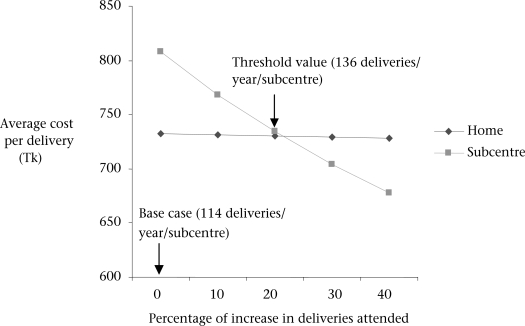
Impact of an increase in deliveries attended by midwives on average cost per delivery

While the average cost of a delivery in the home was insensitive to the number of deliveries (little to no economies of scale), the cost of attending a delivery in the subcentre fell as the number of deliveries in the subcentre increased (Fig. 1). If deliveries in the subcentre increased from 114 to more than 136 deliveries per year, facility-based obstetric care becomes the cheaper option holding other parameters constant.

**Fig. 2.**
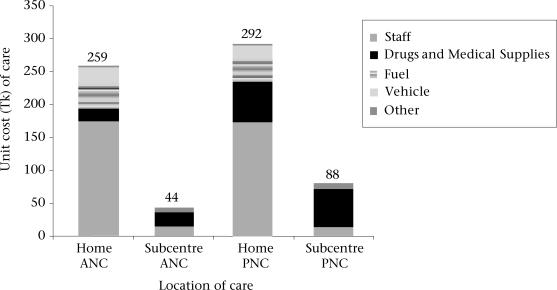
Average costs of antenatal and postnatal care by location

The provision of antenatal and postnatal care in the subcentre involved much less resources than in the home, an estimated difference of between Tk 214 and Tk 217 per visit (Fig. 2). The additional time spent travelling and attending women in the home greatly outweighed the additional equipment and building costs in the subcentre. As the differences in the cost of antenatal and postnatal care provision were large, a threshold analysis was not conducted.

ANC=Antenatal care;
PNC=Postnatal care

## DISCUSSION

Under initial assumptions, it was much cheaper to provide antenatal and postnatal care in a facility than in the home. The cost of providing basic obstetric care in the home and in a facility were similar, although home-based care was slightly cheaper. Deliveries in the home consumed more time but upgrading the subcentres to provide delivery care required substantial investment in equipment and construction. However, if the number of facility-based deliveries increased by more than 22% from the 2001 level (to 136 deliveries per subcentre per month), care in a subcentre would become the cheaper option. Rates of facility-based deliveries have, in fact, been on the increase in Matlab over the years since the study, and 136 deliveries per subcentre per month imply a coverage of 19% which has since been surpassed.

Given that the time spent by a midwife to attend women in the home cannot be used for attending other patients, costs of time associated with a delivery in the home will increase in a direct proportion to the number of deliveries attended. However, the additional time spent by midwives attending deliveries in the home is only an additional cost in so far as it has an ‘opportunity cost’, in terms of other patients who could have been attended. When levels of service-use are low, the opportunity cost of the extra time may be small to zero.

In contrast, as more women come to deliver in the subcentre, the average cost of equipment and construction will fall, making this option cheaper. In this setting, midwives are also able to carry out other activities in the facility, in addition to attending pregnant women. Assuming that midwives are able to attend up to one delivery each per day, they could theoretically handle up to a three-fold rise in the rate of deliveries, suggesting that there is potential for significant economies of scale.

Since safe motherhood programmes aim for maximum coverage of skilled attendance at delivery, facility-based care will be a cheaper and more feasible method for the care provider to meet this objective as demand increases. However, in settings where rates of skilled attendance are very low, home-based care will be cheaper.

Attendance in the subcentre also has economic implications for households. In a separate study published elsewhere, we estimated that there was no significant difference in costs incurred by households for a delivery in the home compared to a delivery in a subcentre (median Tk 130 in the home compared to a subcentre (median Tk 213)) (25). This included transport (median Tk 15), which was minimal as most patients visited the facility on foot. Transport costs were the only cost incurred by households attending a subcentre for antenatal or postnatal care. However, it is important to note that, in the Matlab area, services are provided free of official charge and that costs incurred in basic obstetric facilities within the government sector may be more substantial and serve as a greater barrier to healthcare-seeking (26).

Efficiency is one among other criteria that decision-makers need to consider when prioritizing health expenditure. An important additional issue is acceptability to society and, in this case, households. Evidence from Bangladesh indicates that households can be reluctant to use facilities (27, 28) due to a combination of financial, social and cultural reasons (3). However, the successful transition from home- to facility-based basic obstetric care in Matlab suggests that some of these barriers can be overcome. On the care providers’ side, skilled attendants highlighted some difficulties of conducting deliveries in the home and constraints in their ability to provide quality care, including, for example, lack of supplies and equipment, poor lighting, and pressure to adhere to traditional practices (20). Effectiveness in terms of reductions in mortality and morbidity also needs to be considered. However, the place of delivery has no effect on perinatal mortality (29). The effect on maternal mortality is unknown, although given the overall low-level coverage, an effect would be unlikely. More time is needed to assess the effectiveness of skilled care on mortality as the coverage increases.

Many inputs into the analysis relied on the recall of midwives, which may have been unreliable. This was a major limitation of the study. We were unable to conduct a prospective time-motion study, the gold standard for measuring time spent by health staff (30), due to the change in the medical protocols in 2001. We adjusted for this by conducting a threshold analysis. A further limitation was that the study was conducted in Matlab where the health facilities are more highly funded than the equivalent government facilities. The reason for using this site was that it provided us with a unique opportunity to compare the costs of home- and facility-based care as both policies were implemented sequentially. And while we recognize that the unit costs of care in the ICDDR,B facilities are higher than those in equivalent government facilities, this should not affect the difference in cost between care in the home and in the facility, which is our variable of interest.

The question remains of what is the optimal population coverage and mix for facility- and home-based care. These questions were beyond the scope of this paper and represent an important area for further research.

Since safe motherhood programmes aim for maximum coverage of skilled attendance at delivery, facility-based care is likely to be a cheaper and more feasible method for the care provider as demand rises. In settings where rates of skilled attendance are very low, home-based care will be cheaper. Further research is needed to identify the optimal mix of facility- and home-based care in different settings.

## ACKNOWLEDGEMENTS

This research was funded under the Cooperative Agreement No. 388-A-00-97-00032-00 with the United States Agency for International Development (USAID) and ICDDR,B Grant No. GR-00089. ICDDR,B acknowledges with gratitude the commitment of USAID to the Centre's research efforts. Carine Ronsmans and Jo Borghi were funded by the Department for International Development (DFID), UK.

The authors are grateful to Farhana Khanam and Enamul Hoque for assistance in undertaking this study. They would like to acknowledge the hard work of Rowshan Ara Munni, Ayesha Siddika, Nazneen Rahman, Taniya Yesmin, and Sharmin Sultana Begum who collected data in the field. The authors express gratitude to J**.** Chakraborty, physicians, community health research workers, midwives, clinic attendants, messengers, boatmen/rickshaw-pullers of ICDDR,B and gynaecologists and senior staff nurses of Chandpur District Hospital for their much appreciated cooperation.
